# OUTCOMES OF AN INTEGRATED HEALTH CARE AND COMMUNITY PROVIDER INTERPROFESSIONAL TEAM

**DOI:** 10.1093/geroni/igae098.3021

**Published:** 2024-12-31

**Authors:** Jennifer Drost, Michelle Hughes, Sue Fosnight, Joseph Marchiano, Susan Hazelett, Michele Gareri, Brandi Chrzanowski

**Affiliations:** Summa Health, Akron, Ohio, United States; Direction Home, Akron Canton Area Agency on Aging and Disability, Uniontown, Ohio, United States; Summa Health, Akron, Ohio, United States; Summa Health, Akron, Ohio, United States; Summa Health, Akron, Ohio, United States; Summa Health, Akron, Ohio, United States; Direction Home, Akron Canton Area Agency on Aging and Disability, Uniontown, Ohio, United States

## Abstract

Traditional medical care accounts for 10% of health outcomes, while social determinants of health account for over 60%. To address complex interactions between medical illness, social and behavioral health issues, and medications, effective care requires collaboration between medical and community-based providers. We have developed a collaborative interprofessional model of care between a health system and the local Area Agency on Aging (AAoA) called the Care Management Interdisciplinary Team (CMIT). CMIT care managers (social workers and nurses) complete evaluations of consumers focused on concerns around recent health care utilization (ED visits/hospitalizations), falls, medications, home safety, and behavioral health concerns. Care managers (CM) then present these complex older adults to the team, including geriatric medicine and pharmacy specialists, which develops care plans that are implemented by the CM and the consumer’s primary care physician. CM presented cases between January-December 2023 and worked to follow up at 3 months. Eighty-one consumers were presented to the CMIT team over a 1-year period. A 3-month follow up is available for 44 consumers (54%). Of those with 3-month follow ups, at baseline, 10 (23%) consumers had recent health care utilization episodes, 17 (39%) had concerns about falls, 17 (39%) had concerns about medications, 21 (48%) had home safety concerns, and 10 (23%) had behavioral health concerns. At the 3-month follow up, 5 consumers had improved health care utilization, 6 fewer had falls, 5 had medication changes, 13 had improved home safety concerns, and 7 had improved behavioral health concerns.

